# The Comparative Study on Expression of SIRT1 Signal Transduction by Xuefuzhuyu Capsule

**DOI:** 10.1155/2014/537014

**Published:** 2014-07-08

**Authors:** Fei Teng, Guangxi Li, Zhangjing Liu, Liangdeng Zhang, Kuiwu Yao

**Affiliations:** Guang'anmen Hospital, China Academy of Chinese Medical Sciences, Beijing 100053, China

## Abstract

The Xuefuzhuyu capsule (XFZY) is widely used for the treatment of ischemic heart disease (IHD) in China. We previously demonstrated that XFZY could reduce apoptosis in Sprague-Dawley rat cardiomyocytes with the similar effect of resveratrol (Res) Hori et al. (2013), although its molecular mechanism underlying this protective effect is still unclear. Silent information regulator of transcription 1 (SIRT1) had been demonstrated to be responsible for cardioprotection against ischemia-reperfusion injury via long-term transcriptionally regulatory mechanism Braunersreuther and Jaquet (2012). Therefore, in the present study, we aimed to test if XFZY might contribute to the protection of ischemic myocardial cells induced by ischemia through SIRT1-mediated signal transduction pathway by using electron micrograph, RT-PCR assay, and western-blot test. All the result showed that the target genes of SIRT1 pathway including P53, NF-kB, FOXO1, FOXO3, and FOXO4 were significantly downregulated to SIRT1, suggesting that apoptosis pathway might transcriptionally be regulated to SIRT1. In addition, the expression level of SIRT1 was significantly increased by XFZ, it might prove that SIRT1 is the target of XFZY working on ischemia heart disease. Our findings supported that XFZY might function to protect myocardial cells and reduce myocardial injury though SIRT1 signaling pathway and has the same pharmacological effect with Res.

## 1. Introduction

Ischemic heart disease (IHD) is one of the most serious human disorders leading to long-term reduced mobility and high mortality. It induces irreversible myocardial damage despite relieving the myocardial ischemia, which in turn leads to cardiac remodeling characterized by dilation of the left ventricle (LV) and reduced contractility. In this disorder, ischemia reperfusion had been found to be a major cause of myocyte necrosis and apoptosis [1], and ischemia/reperfusion (I/R) injury remains the major cause of heart failure and arrhythmia. Although many interventions alleviating the extent of myocardial injury in animal models of I/R have been tested in patients, thus far none of them have exhibited definitive advantages over the control, suggesting that a novel mechanism of intervention is needed [2].

Elucidating the mechanisms mediating aging and controlling the lifespan of organisms is an important theme in modern biology, and it is widely accepted that the myocardial oxygen delivery that is insufficient in relation to myocardial oxygen demand is a prominent factor in triggering the events that ultimately result in cardiomyocyte death [3–5]. A recent finding suggested that cardiac SIRT1 is significantly upregulated in response to oxidative stress and because of that it can prolong the lifespan of cardiac myocyte and recover cardiac myocyte function [6]. Sirtuin 1 (SirT1) belongs to the sirtuin family of nicotinamide adenine dinucleotide NAD-dependent protein deacetylases, which are involved in a variety of cellular functions such as gene silencing, heterochromatin formation, cell survival, metabolism, and development [7–9] and its activation is considered beneficial for metabolic, neurodegenerative, and inflammatory diseases and to augment longevity, as well as protective effectiveness during ischemia/reperfusion processes and neurotic generation [10].

A variety of studies had demonstrated that Chinese medicine has specific protective effects against ischemia episodes in heart and brain. Zhu et al. investigated that icariin (ICA) could protect brain from ischemic injury by increasing expression level of SIRT1 and PGC-1a during ICA's neuroprotection against ischemia [11]. Other studies had also shown that Chinese herbs or traditional medicine monomers are cardioprotective against ischemia, but related mechanism is still unclear. However, few studies focused on the role of Chinese herbal compound for myocardial ischemia [12, 13]. Resveratrol (Res) is known to improve treatment outcome after ischemic episodes in heart and require SIRT1 to mediate ischemic protection to increase lifespan [14]. The Xuefuzhuyu capsule (XFZY) is widely used for the treatment of ischemic heart disease (IHD) in China with the protection of cardiomyocytes from injury by ischemia, but the molecular mechanism underlying this protective effect is still unclear.

In this study, we observed morphological changes of ischemic myocardium of Sprague-Dawley rats by electron micrograph. To investigate the action mechanism of XFZY on antiapoptosis in IHD and to identify a promising strategy for the treatment of ischemia-induced injury, we also examined the expression levels of SIRT1 as well as its target genes and the protein in ischemic myocardium by RT-PCR assay and Western-blot analysis.

## 2. Materials and Methods

### 2.1. Experimental Materials

#### 2.1.1. Animals

Adult male Sprague-Dawley rats (200 ± 5 g) were from the Experimental Animal Center at the Xiyuan Hospital China Academy of Chinese Medical Sciences. Five rats were kept in each cage, and the rats were conditioned for one week at room temperature (23 ± 1°C), with a constant humidity of 55 ± 5%, under a cycle of 12 h of light/dark, and had free access to food and tap water.

#### 2.1.2. Drugs

The Xuefuzhuyu capsules (XFZY) were purchased from Tianjin Hong Ren Tang Pharmaceutical Co., Ltd. The drug compositions are* Rehmannia*, Peach kernel, Safflower, Chinese Angelica, Licorice, Radix Paeoniae Rubra, bellflower, Fructus aurantii,* Bupleurum*, Chuanxiong, and* Achyranthes bidentata*. The powder of resveratrol (Res) with 100 mg/bottle was purchased from Sigma Biotech Company.

#### 2.1.3. Instruments

The instruments used were RM-6000-type eight polygraph (NIHON KOHDEN XDH-3B), ECG machine (Shanghai Medical Electronic Instrument Factory), DH-150 animal-artificial respiration machine (Zhejiang University Medical Instrument Factory), 7900HT quantitative PCR instrument (Applied Bio systems Inc.), Gel image analyzer Image Master VDS (United States Pharmacia Biotech), High-speed refrigerated centrifuge (Germany BACKMAN Company), UV spectrophotometer (Germany BACKMAN the DU640-type), and Electrophoresis instrument (Beijing sixty-one Instrument CYY-III-5 type).

### 2.2. Experimental Methods

#### 2.2.1. Grouping Method

**Figure 1:**
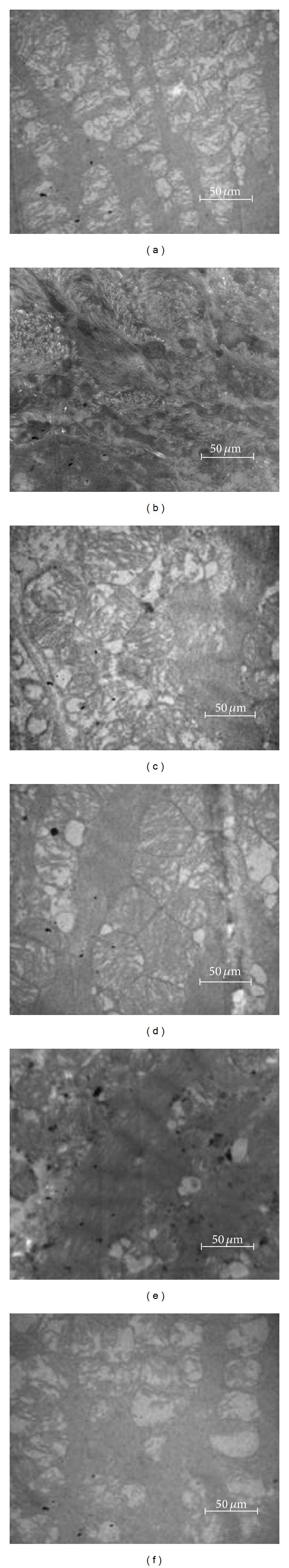
(a) Normal group. (b) Ischemia group. (c) Sham-operated group. (d) Res group. (e) XFZY group. (f) L-NAME group.

Sprague-Dawley rats were screened by electrocardiogram (ECG) before experiment. The normal ones were divided randomly into 6 groups: normal group, sham-operated group, ischemia group as negative control, XFZY-treated group, Res-treated group, and L-NAME group, with 10 of rats in each group (Figure 1). Except for normal and sham-operated groups, the rest of rats were prepared for the model of myocardial ischemia. Sham-operated rats treated with sham operation.

#### 2.2.2. Animal Experimental Model

We ligate the left coronary artery of rats; the basic approach is to perform a left thoracotomy and secure a ligature around the intramyocardial portion of the artery that lies just ventral to the left atrium. Both male and female rats have undergone coronary artery ligation, and while the most common species is adult Sprague-Dawley rats, our laboratory has also infarcted Fischer-344 and Brown Norway Fischer-344 cross rats. The approach used in our laboratory is as follows. After induction of anesthesia with acepromazine maleate 50 mg/kg, xylazine 5 mg/kg, and ketamine He1 50 mg/kg intraperitoneally, a left anterior thoracotomy is performed under sterile conditions. The heart is expressed through the incision and a 7-0 synthetic ligature is secured snugly around the proximal left anterior coronary artery. The lungs are inflated to reduce the pneumothorax, and the muscle layer and skin are closed separately. Postoperative analgesia is provided with acetaminophen (67 rag/L) in the drinking water. An acute survival rate of approximately 50% is generally achieved. Other variations on this basic approach are to use endotracheal intubation with ventilator support so as to have more time to perform the ligation and to treat rats with perioperative lidocaine to decrease the incidence of ventricular tachycardia and fibrillation.

#### 2.2.3. Administration

After modeling, rats in XFZY groups were treated orally with 30 mg/kg of XFZY 1/d. Rats in normal group, sham-operated group, and ischemia group were treated with equal volume of saline, 1/d. Rats in Res group were treated with 10 mg/kg of Res by sublingual intravenous administration, 1/d for 10 days. Rats in L-NAME group were intraperitoneal injected before modeling and on the day of the experiment, 2 mg of L-NAME for each 1/d.

#### 2.2.4. Specimen Collection for Further Assays

After intraperitoneal anesthesia by 10% chloral hydrate, each rat heart exposed for thoracotomy was infused with 200–300 mL of saline and electron microscopy fixative (1% paraformaldehyde and 2.5% glutaraldehyde), respectively, from the left ventricular rapidly, and was sustained for 30–60 minutes until the upper limbs, neck, and lower extremity of the rat were stiff. For electron micrograph, the left ventricular anterior myocardial above the ligature was cut into 1 × 1 × 1 mm pieces. Subsequently, specimens were prefixed with 2.5% of glutaraldehyde and fixed with 1% of osmium tetroxide, followed by dehydrated with acetone. For RT-PCR assay, the myocardial above the ligature was cut into 4 mm thick pieces.

#### 2.2.5. Quantitative Real-Time QRT-PCR Assay

L-NAME was purchased from Alexis Company. Trizol was purchased from Invitrogen, Revert Aid TM First Strand cDNA Synthesis Kit was purchased from Fermentas and SYBR Green PCR Master Mix was purchased from Applied Biosystems.

Primers were designed using Primer Premier 5.0 software and their sequence was as follows: Bcl-2 (GenBank Accession number L14688).

Real-time PCR was performed using a LightCycler (Roche Diagnostics, Indianapolis, IN, USA) and individual PCRs were carried out in 96-well optical reaction plates according to the manufacturer's instructions. Briefly, the PCR was carried out in a 25 *μ*L final volume containing the following: 12.5 *μ*L of 2x SYBR Green I master mix, 0.5 *μ*L of 10 *μ*M each primer, 2 *μ*L of 1 *μ*g/*μ*L cDNA template, and 9.5 *μ*L of 0.1% diethylpyrocarbonate-treated water. The PCR conditions were as follows: initial denaturation at 95°C for 10 s, followed by 40 cycles of denaturation at 95°C for 5 s, annealing at 59°C for 15 s, and extension at 72°C for 15 s. Fluorescent product was measured by a single acquisition mode at 72°C after each cycle. The quantification of the target gene expression was conducted according to a published method [12]. Briefly, standard curves for target genes and the housekeeping gene were constructed using their DNA isolated using a DNA extraction kit with serial dilutions (10-fold dilution). The standard curve samples were included in each PCR. Standards for both target and internal DNA were defined to contain an arbitrary starting concentration because no primary calibrators exist. Hence, all calculated concentrations are relative to the concentration of the standard. Negative controls (replacement of cDNA with water) were run with each set of reactions. To distinguish the specific PCR products from nonspecific products and primer dimers, a melting curve was obtained after amplification by holding the temperature at 65°C for 15 s followed by a gradual increase in temperature to 95°C for 50 s. The signal acquisition was set at “continuous” mode for the detection. GAPDH mRNA level was used as an internal quantitative control, and the level of each target gene transcript was normalized on the basis of GAPDH mRNA content.

The relative expression level was determined by 2^−ΔΔCt^ method according to the following formula: ΔCt (target gene) = target gene Ct − actin Ct; ΔΔCt = ΔCt (target gene) − ΔCt (standard value); the copy of the target gene is 2^−ΔΔCt^.

RNA was determined by ultraviolet spectrophotometer at *λ*260 nm/*λ*280 nm with the range between 1.8 and 2.0. The melting curve of PCR amplification product was substantially a single temperature peak without distortion.

#### 2.2.6. Western-Blot Analysis

After treatment, cells were washed twice with ice-cold PBS and then lysed on ice in extraction buffer containing 50 mMTris-base (pH 7.4), 100 mM NaCl, 1% NP-40, 10 mM EDTA, 20 mM NaF, 1 mM PMSF, 3 mM Na_3_VO_4_, and protease inhibitors. The homogenates were centrifuged at 12,000 g for 15 min at 4°C. Supernatant was separated and stored at −80°C until use. Protein concentration was determined by using the BCA protein assay kit (Pierce Biotechnology, Rockford, IL, USA). Protein samples (50 *μ*g) were separated by 10% SDS-polyacrylamide gel electrophoresis and then transferred to nitrocellulose membranes. After being blocked with 5% nonfat milk in Tris-buffered saline containing 0.1% Tween 20 (TBST) for 1 h at room temperature, transferred membranes were incubated overnight at 4°C with different primary antibodies (Table 2). After three washes with TBST, the membranes were incubated with horseradish peroxidase-conjugated secondary antibodies (1 : 5000) in TBST with 3% nonfat milk for 1 h at room temperature. After repeated washes, membranes were reacted with enhanced chemiluminescence reagents (Amersham Pharmacia Biotech, Piscataway, NJ, USA) for 3 min and visualized with X-ray films (Kodak X-Omat, Rochester, NY, USA). The films were scanned and the optical density of the bands was determined using Optiquant software (Packard Instruments). The expression levels of SIRT1, P53, FOXO family, and NF-*κ*B of treated cultures were compared with those of untreated control cultures. Normalization of results was ensured by running parallel Western blots with *β*-actin.

All experimental procedures were conducted in conformity with institutional guidelines for the care and use of laboratory animals in Guang' anmen Hospital, China Academy of Chinese Medical Sciences, Beijing, and conformed to the National Institutes of Health Guide for Care and Use of Laboratory Animals (Publication number 85-23, revised 1985).

## 3. Results

### 3.1. The XFZY Treatments Restored Injury of Myocardial Ischemia in the Rat Models with Electron Micrograph

In both ischemia and L-NAME groups, there was almost no complete structure of muscle fiber observed and the myocardial cells were replaced by certain amount of clutter collagen fibers, mitochondrial swelling, vacuolization and cristae disorganized. The nuclear membrane was broken with uneven matrix, vacuolization, widened week gap, and fuzzy structure of intercalated disc.

The results in both normal and Res groups showed that the myocardial cells arranged in neat rows and the gap was clear with a small amount of collagen fibers. The sarcomere was clear with normal structures of intercalated disc and mitochondrial showing membrane integrity. Nuclear membrane and organelles were intact with no inflammatory cell infiltration observed.

In XFZY group, local myofilament was broken with the clear structure of sarcomere, while length of sarcomere was uniform and swelling of mitochondria was mild with the neat structure of crest. The nuclear matrix was mildly cavitative and nucleoli were visible with normal structure of intercalated disc, showing similar images to that of Res group.

### 3.2. The Impact of Interventions of XFZY on SIRT1 Pathway in RT-PCR Assay

**Figure 2:**
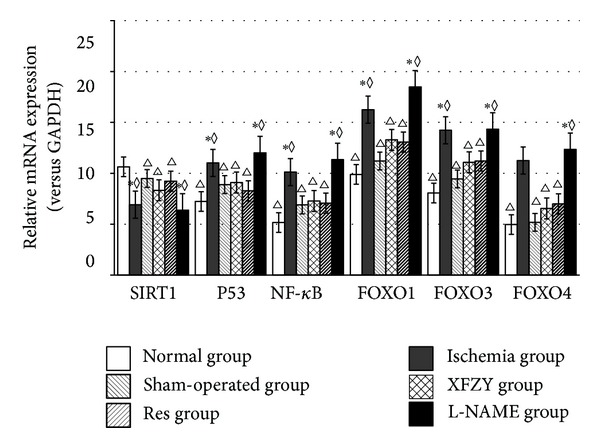


Compared with the normal group, the expression levels of SIRT1 had significant difference in ischemia group and L-NAME group. The expression levels of P53, (NF)-kappa B, FOXO1, FOXO3, and FOXO4, as SIRT1 target genes (Table 1), were significantly different with ischemia group and L-NAME group too. XFZY was upregulated with the SIRT1, while downregulated with P53, (NF)-kappa B, FOXO1, FOXO3, and FOXO4. Compared with the ischemia group, the expression levels of SIRT1 had significant difference compared with normal group, sham-operated group, XFZY group, and Res group, while the expression levels of P53, (NF)-kappa B, FOXO1, FOXO3, and FOXO4 were significantly different with ischemia group and L-NAME group. The expression of SIRT1, P53, (NF)-kappa B, FOXO1, FOXO3, FOXO4, and L-NAME in XFZY group were very similar with Res group and that might be the reason for why XFZY could treat IHD. (See Figure 2 and Tables 3, 4, 5, 6, 7, and 8).

### 3.3. The Impact of Interventions of XFZY on SIRT1 Pathway in Western-Blot Analysis

**Figure 3:**
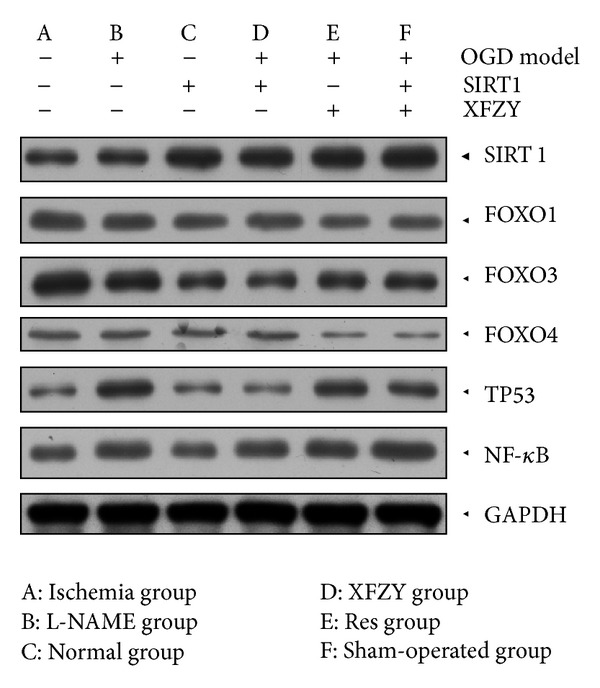


**Figure 4:**
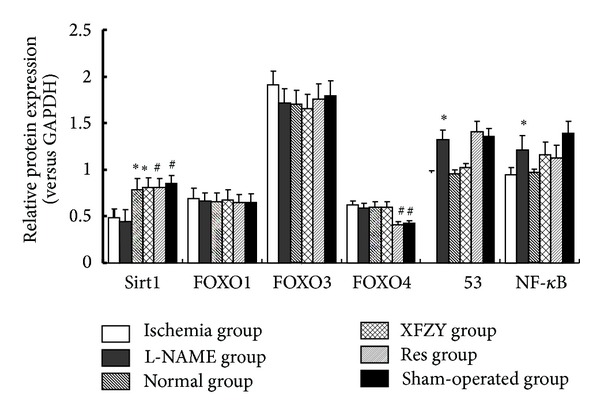


(See Figures 3 and 4).

## 4. Discussion

In this study, we used Chinese medicine XFZY to treat IHD model rats, observed morphological changes of ischemic myocardium by electron micrograph, and examined the mRNA expression levels of SIRT1 and its target genes by RT-PCR assay, and then we examined the relative protein by western-blot analysis. Among those 6 groups, Res is well known to improve the outcome after ischemic episodes and requires SIRT1 to mediate ischemic protection to increase lifespan [14]; therefore, we used Res as a drug positive control to investigate the action mechanism of XFZY. We also designed L-NAME group as the negative control group.

The results from electron micrograph in normal and Res groups apparently showed the best image of myocardium that the cells arranged in neat rows, the gap was clear with a small amount of collagen fibers, sarcomere was clear; structure of intercalated disc and mitochondrial were normal. In the ischemia and L-NAME group, the damages of myocardium with almost no complete structures of muscle fiber were shown. The image in the L-XFZY group was even worse than the ischemia group with local myofilament broken. Abbatea and so forth had observed ischemic myocardium of New Zealand rabbits by electron microscopy and found that the changes of mitochondria and myofibrillar were highly correlated with myocardial cell apoptosis [15]. Others also drew similar conclusions by researches on characterization of cardiomyocyte apoptosis in ischemic heart disease, and the changes of mitochondrial were most obvious [16, 17].

Previous study proved that SIRT1 activation elicited resistance to oxidative stress via regulation of transcription factors and coactivators such as FOXO 1, 3, and 4, Hif-2a, and NF-*κ*B [17, 18]. P53 is an important factor regulated with myocardial apoptosis, whose role is mainly through the activation of the renin-angiotensin system. Lots of researches supported that when myocardial ischemia happened, SIRT1 could reduce activity of P53 through deacetylation and suppress the cardiomyocyte apoptosis [19–21]. NF-*κ*B is a heterodimeric protein, controlling the expression of the cell survival gene. Wang and so forth revealed that SIRT1 inhibits transcription of RelA/p65 subunit by deacetylation of NF-*κ*B and reduces the generation of oxygen free radicals so as to inhibit the main pathological factors which promote atherosclerosis and cardiovascular disease [22]. FOXO family is a kind of important acting factor of SIRT1, and it was generally believed that SIRT1 could promote activity of FOXO1 to control cellular oxidative stress response by making FOXO1 deacetylation [23]. Recent research found that SIRT1 has a similar role of FOXO3 and FOXO4 [24]. The study on signaling pathway of ischemic heart disease showed that the SIRT1-FOXO pathway is a major signaling pathway for inhibition of myocardial apoptosis [25]. Also, our research findings verified those previous studies.

With the in-depth research on pathogenesis of ischemic heart disease, to protect undead ischemic myocardial cells is becoming the most valuable therapeutic strategy which should be taken in preventing myocardial apoptosis. SIRT1 plays an important role in a number of human physiological and pathological processes, including chronic inflammation, cancer, diabetes, and longevity, especially in ischemic injury [25–29]. As a regulatory protein deacetylase, SIRT1 has certain cardioprotection including activation of endothelial nitric oxide synthase, insulin receptor signalling, heart embryonic development, and autophagy [30, 31]; therefore, in this study we chose SIRT1 as a key point. Although we initially validated the impact of interventions of XFZY on SIRT1 signal transduction pathway, the mechanism of its action needs to be further elucidated through the SITR1 pathway by using both* in vitro* and* in vivo* models in our future studies.

In summary, XFZY had shown the bioactivities of antimyocardial apoptosis and cardioprotection in this study. The mechanism of this drug action might be through the impact of SIRT1-mediated signal transduction pathway on cardiomyocytes of ischemic heart disease, which provides us with a promising insight for the intervention of TCM on IHD.

## Figures and Tables

**Table 1:** 

Target gene	The primer sequences	Amplification length (bp)
P53	Upstream: 5′-GCAGTTCCTCTTCCTGCAGTACTC-3′;	241
	Downstream: 5′-AAC CAGACCTCAGGCGGCTCATAG-3′	
NF-*κ*B	Upstream: 5′-CGATCTGTTTCCCCT CATCT-3′	175
	Downstream: 5′-ATTGGGTGCGTCTTAGTGGT-3′	
FOX01	Upstream: 5′-AACCAGCTCAAAT GCTAGTACCATC-3′	198
	Downstream: 5′-CAGAAGGTTCTCCATGTTTTTCT GGA-3′	
FOX03	Upstream: 5′-TACACGGCTTGCTTACGG-3′	423
	Downstream: 5′-GGG TTC AGA ACG AAG GGA CT-3′;	
FOX04	Upstream: 5-TCATCAGCCAGGCCATTGAA-3′	174
	Downstream: 5′-TGCTGTGCAAAGACAGGTTGTG-3′;	
Sirt1-2	Upstream: 5′-TTTCAGAACCACCAAAGCG-3′	206
	Downstream: 5′-TCCCACAGGAAACAGAAACC-3′	
*β*-Actin	Upstream: 5-GAG ACC TTC AAC ACC CCA GCC-3	263
	Downstream: 5-AAT GTC ACG CAC GAT TTC CC-3	

**Table 2:** The information of primary antibodies.

Antibody	Company	Species	Dilution factor
Sirt1	Epitomics	Rabbit	1 : 2500
FOXO1	Epitomics	Rabbit	1 : 1000
TP53	Epitomics	Rabbit	1 : 2000
FOXO3	Epitomics	Rabbit	1 : 2000
FOXO4	Epitomics	Rabbit	1 : 2000
NF-kB	Epitomics	Rabbit	1 : 2000
GAPDH	Sigma	Goat	1 : 8000
Anti-rabbit	Sigma	Goat	1 : 8000

**Table 3:** Expression of SIRT1 by quantitative real-time RT-PCR.

Group	*n*	SIRT1
Normal group	10	10.64 ± 2.01^△^
Ischemia group	10	6.92 ± 1.21^∗◊^
Sham-operated group	10	9.49 ± 1.31^△^
XFZY group	10	8.33 ± 2.14^△^
Res group	10	9.22 ± 2.29^△^
L-NAME group	10	6.38 ± 2.93^∗◊^

Compared with the normal group, *P < 0.05; compared with the ischemia group, ^△^ *P* < 0.05; compared with the XFZY group, ^◊^ *P* < 0.05.

**Table 4:** Expression of P53 by real-time PCR.

Group	*n*	P53
Normal group	10	7.23 ± 1.31^△^
Ischemia group	10	11.02 ± 1.78^∗◊^
Sham-operated group	10	8.89 ± 2.63^△^
XFZY group	10	9.09 ± 1.65^△^
Res group	10	8.27 ± 4.29^△^
L-NAME group	10	12.01 ± 2.18^∗◊^

Compared with the normal group, *P < 0.05; compared with the ischemia group, ^△^ *P* < 0.05; compared with the XFZY group, ^◊^ *P* < 0.05.

**Table 5:** Expression of nf-*κ*B by real-time PCR.

Group	*n*	nf-*κ*B
Normal group	10	5.16 ± 2.23^△^
Ischemia group	10	10.12 ± 1.89^∗◊^
Sham-operated group	10	6.89 ± 2.15^△^
XFZY group	10	7.28 ± 1.35^△^
Res group	10	7.07 ± 1.29^△^
L-NAME group	10	11.34 ± 2.09^∗◊^

Compared with the normal group, *P < 0.05; compared with the ischemia group, ^△^ *P* < 0.05; compared with the XFZY group, ^◊^ *P* < 0.05.

**Table 6:** Expression of FOXO1 by real-time PCR.

Group	*n*	FOXO1
Normal group	10	9.89 ± 1.83^△^
Ischemia group	10	16.25 ± 2.07^∗◊^
Sham-operated group	10	11.21 ± 1.59^△^
XFZY group	10	13.28 ± 1.35^△^
Res group	10	13.07 ± 1.09^△^
L-NAME group	10	18.48 ± 2.79^∗◊^

Compared with the normal group, *P < 0.05; compared with the ischemia group, ^△^ *P* < 0.05; compared with the XFZY group, ^◊^ *P* < 0.05.

**Table 7:** Expression of FOXO3 by real-time PCR.

Group	*n*	FOXO3
Normal group	10	8.06 ± 1.32^△^
Ischemia group	10	14.23 ± 2.09^∗◊^
Sham-operated group	10	9.45 ± 1.15^△^
XFZY group	10	11.08 ± 0.97^△^
Res group	10	11.21 ± 0.67^△^
L-NAME group	10	14.34 ± 2.13^∗◊^

Compared with the normal group, *P < 0.05; compared with the ischemia group, ^△^ *P* < 0.05; compared with the XFZY group, ^◊^ *P* < 0.05.

**Table 8:** Expression of FOXO4 by real-time PCR.

Group	*n*	FOXO4
Normal group	10	4.96 ± 1.13^△^
Ischemia group	10	11.25 ± 0.89^∗◊^
Sham-operated group	10	5.18 ± 1.45^△^
XFZY group	10	6.54 ± 1.65^△^
Res group	10	6.99 ± 1.19^△^
L-NAME group	10	12.34 ± 1.09^∗◊^

Compared with the normal group, *P < 0.05; compared with the ischemia group, ^△^ *P* < 0.05; compared with the XFZY group, ^◊^ *P* < 0.05.
